# Promoting multilingualism in dementia research, prevention, and discourse

**DOI:** 10.1590/1980-5764-DN-2024-0201

**Published:** 2024-12-02

**Authors:** Timothy Daly

**Affiliations:** 1 Bioethics Program, FLACSO Argentina, Buenos Aires, Argentina.; 2 Sorbonne Université, Science Norms Democracy UMR 8011, Paris, France.

Dear Editor,

A *lingua franca* in science and medical practice is essential for the rapid dissemination of scientific discoveries and in public health emergencies. At the beginning of the 20^th^ century, French and German were the most important languages, but English is currently the most widely used. For practical reasons, it makes sense to choose a single language, even though this leads to inequities^1^. English is arguably the most appropriate candidate, due to the importance and number of publications in this language.

However, journals such as *Dementia & Neuropsychologia* should be commended for promoting multilingualism on their website, not only by offering article titles and summaries (in English/Portuguese), but also by providing tests or questionnaires adapted to the language of the country to be published on the journal's website, making them readily available for use in research or clinical practice, with Spanish soon to be available for such purposes. Such editorial initiatives are crucial because, as we move away from the short-term benefits of monolinguistic communication in science and medicine, over-reliance on English alone in dementia research leads to various long-term issues.

Languages are value-laden, and dependence on English in the academic space increases the likelihood of value-laden language becoming a monolith^2^. This has direct implications for dementia research. For instance, Hatahet and colleagues recently highlighted the "lack of culturally valid tests for speakers of understudied languages,"^3^ due to the major dominance of languages like English in dementia research. In other words, over-reliance on English reduces the international medical community's ability to diagnose this condition.

Beyond issues with diagnosis, the worldwide dominance of English is also an issue from the perspective of dementia prevention, which the dementia research community should address. Indeed, there is well-established research on the protective effects of bilingualism, which may increase cognitive reserve^4^. However, the prevalence of "linguistic colonialism" globally, which favors the use of English in professional, personal, and even cultural interactions^5^, means that millions of English speakers may never actually master another language enough to use it in their daily lives, and many learners of English will never need a third language beyond their mother tongue and English. If it is true that learning additional languages is protective for the brain, this represents a significant lost opportunity for improving brain health worldwide. While the outdated "monolingual mindset" may consider English proficiency to be an outright privilege in itself, data from the English-speaking regions suggest that multilingual members of society who also master English tend to have better outcomes in education, employment, and income^6^.

There may be two mechanisms by which multilingualism is protective: both direct brain stimulation^7^, and the enhanced social mobility of people who speak more than one language. Therefore, in addition to supporting the idea that "linguistic diversity should be considered in the development of cognitive tests"^3^ through inclusive editorial policies within dementia research, the dementia researcher community should also advocate for multilingualism within broader aspects of culture so as to promote access to brain health for all.
